# Virtual Reality-Assisted Cognitive Behavioral Therapy for Anxiety Disorders: A Systematic Review and Meta-Analysis

**DOI:** 10.3389/fpsyt.2021.575094

**Published:** 2021-07-23

**Authors:** Jinlong Wu, Yi Sun, Gongwei Zhang, Zhenhui Zhou, Zhanbing Ren

**Affiliations:** ^1^Shenzhen University, Shenzhen, China; ^2^College of Physical Education, Jilin University, Changchun, China; ^3^Department of Radiology, Shenzhen Children's Hospital, Shenzhen, China; ^4^Shenzhen Children's Hospital, Shenzhen, China

**Keywords:** cognitive behavioral therapy, virtual reality, anxiety disorder, meta, systematic review

## Abstract

**Objective:** We aim to explore the effectiveness of virtual reality-assisted cognitive behavioral therapy (VRCBT) in the treatment of anxiety and depression in patients with anxiety disorders. We further compare the therapeutic effect of VRCBT with that of standard cognitive behavioral therapy (CBT), as well as investigate the long-term efficacy of VRCBT.

**Methods:** As of March 3, 2020, a total of four databases (Web of Science, PubMed, PsycINFO, and Scopus) were retrieved, and two researchers independently conducted literature retrieval and research selection and performed data extraction. Methodological quality assessment was performed using the Cochrane risk of bias tool and Grading of Recommendation, Assessment, Development, and Evaluation tool (GRADE).

**Results:** A total of 11 studies were included (*n* = 626; range, 25.3–43.8), and six randomized controlled trials were quantitatively analyzed. The main outcome was anxiety and depression, and the secondary outcome was the withdrawal rate and long-term effects. Meta-analysis showed that the therapeutic effect of VRCBT on anxiety was better than that of the waiting list group (WLG) (SMD = −0.92; 95% CI: −1.34, −0.50; *p* = 0.005, *I*^2^ = 66%, *n* = 276), while the therapeutic effect of VRCBT on anxiety was similar to that of standard CBT treatment (SMD = −0.26; 95% CI: −0.50, −0.01; *p* = 0.77, *I*^2^ = 0%, *n* = 150). We further found that the therapeutic effect of VRCBT on depression was better than that of the WLG (SMD = −1.29; 95% CI: −2.26, −0.32; *p* = 0.09, *I*^2^ = 58%, *n* = 74), while the effect of VRCBT was similar to that of standard CBT (SMD = −0.30; 95% CI: −0.67, −0.07; *p* = 0.39, *I*^2^ = 1%, *n* = 116). Of the five studies that reported withdrawal rates of patients during the VRCBT and CBT treatment process, the withdrawal rates of the VRCBT group and CBT group did not reach statistical significance (OR = 0.70, 1.48, *p* > 0.05); only two studies reported the long-term effectiveness of VRCBT in anxiety and depression on patients with anxiety disorders.

**Conclusion:** VRCBT treatment has a specific positive effect on patients with anxiety disorders (anxiety and depression). Compared with standard CBT, similar therapeutic effects can be achieved in the treatment of anxiety disorders. However, limited randomized controlled trials were included, requiring that these results be treated with caution.

## Introduction

Anxiety disorder is the most common mental illness and is characterized by excessive anxiety, fear, and corresponding behavioral disturbance (1). Depressive symptoms often accompany patients with anxiety disorders, and some studies have shown that the anxiety symptoms of patients with depression are more serious (2). A meta-analysis of the results of epidemiological surveys in 44 countries has shown that the prevalence of anxiety disorders is about 7.3% worldwide (2). These diseases are closely related to high social costs (3), social–psychological function, and reduced quality of life (4, 5). Generally, mental illnesses, such as anxiety disorders, are often difficult to detect in time.

Cognitive behavioral therapy (CBT) is an effective method for treating anxiety disorders (6, 7). It is administered in the form of individual or group settings, and therapists are able to reduce patients' psychological pain by changing their way of thinking and behavior (8). Exposure is viewed as a fundamental part of CBT for anxiety disorders, which is very problematic for the treatment process. As part of the therapy, patients can be asked to make a public speech in front of a group of people or be put in awkward and uncomfortable situations (9). Moreover, the situational factors involved in this type of therapy are difficult to control, often making it difficult for therapists to implement CBT (10). Simultaneously, when conducting imaginal exposure, the effects can be limited by the patient's imagination and cognitive function (11).

These weaker aspects of these standard CBTs can be augmented by virtual reality (VR). As a newly developing intervention method, VR has gradually become an adjacent therapy to various disease treatments, such as for cerebral palsy, depression, and Parkinson's disease. VR technology is a human–computer interaction technology based on multisensory perception and has the advantage of creating a sense of immersion while providing timely feedback based on personal performance (11–14). VR was initially widely used in specific phobias, such as arachnophobia (fear of spiders) and aerophobia (fear of flying), and has expanded to more complex anxiety disorders, such as in the treatment of obsessive–compulsive disorder and acrophobia (fear of heights) (10, 15).

For patients with anxiety disorders, virtual exposure can provide multiple advantages compared to standard CBT. In standard CBT, real exposure can be difficult and potentially dangerous (as in the case of posttraumatic stress disorder, for example), or the treatment cost may be too high (phobias in acrophobia) (10, 15). In VRCBT treatment, the therapist no longer needs the patient to carry out exposure therapy in reality, but VR can realize the exposure therapy according to the patient's condition. It can create realistic virtual environments based on different anxiety disorders, accurately shifting to the patient's immersive environment, and expose the virtual environment in different stages according to the needs of the disease. VR therefore allows therapists and patients to fully control the stimulus and exposure. During the course of treatment, therapists can view patients' environment being seen on the screen, and simultaneously observe patients' discomfort and adjust the degree of stimulation (10, 16). Such VR exposure treatment can maximize treatment effectiveness under the condition of complete confidentiality (17) and engage patients to participate in treatment through virtual scenes or *via* direct communication with psychotherapists about potentially uncomfortable subjects.

Because of these advantages, VR therapy has been developed and applied to evaluating and treating various psychological issues. Previous studies have examined the therapeutic effects of using VR therapy alone as well as with traditional treatment options. Powers et al. (18) found in a meta-analysis that VR group patients showed improved effects compared to reality exposure therapy. A further meta-analysis (19) found that VR patients showed moderate to large-scale effects in overall subjective pain, cognitive change, and behavioral measurements of physiological indicators compared to traditional anxiety disorder treatments. It therefore appears that compared to the traditional treatment scheme, VR can achieve similar therapeutic effects. To this effect, a study found that VRCBT seemed to be more conducive to the treatment of anxiety disorders compared to the traditional exposure therapy of CBT (20). In the published meta-analysis, there is no research on the difference between VRCBT and CBT.

In the current study, we conducted a detailed meta-analysis intended to further elucidate the potential benefits of VR therapy. By collecting randomized controlled trials using VRCBT to treat anxiety disorders, we explored the effects of VRCBT on anxiety and depression in patients with anxiety disorders. We further examined the differences in therapeutic effects between VRCBT and standard CBT as well as the long-term effect of VRCBT in order to provide guidance for clinical psychotherapists treating patients with an anxiety disorder.

## Methods

This study was conducted according to the guidelines in the Preferred Reporting Items for Systematic Reviews and Meta-Analyses Protocols (PRISMA-P) (21).

### Literature Search

Electronic searches were conducted using the Web of Science, PubMed, PsycINFO, and Scopus databases from inception through March 3, 2019, to identify all relevant published articles, The search terms included (virtual reality) and (behavior^*^ therap^*^ or cognitive therap^*^ or cognitive behavior^*^ therap^*^) and (GAD or generalized anxiety disorder or OCD or obsessive–compulsive disorder or social phobia social or anxiety disorder or specific phobia or simple phobia or PTSD or posttraumatic stress disorder or acute stress disorder), and we searched in the database by “subject” or “title/abstract.” To include all relevant research, we further, manually searched relevant research from recently published meta-analyses and review articles.

### Eligibility Criteria

Article screening was independently carried out by two researchers based on the title and abstract, taking into account the research on the therapeutic effect of VRCBT on patients with anxiety disorders. The procedure was intended to eliminate duplicate, irrelevant, and review literature, and then further refine the screening according to the full-text inclusion criteria: (1) Literature type: All the controlled trials compared the effects of VR combined with CBT on patients with anxiety disorders. All the studies were published literature, excluding conference abstracts, and case studies, regardless of whether allocation concealment and blindness were used. (2) Research subjects: The experimental subjects were between 18 and 65 years old, and the structured diagnosis was determined by experienced therapists, which conformed to the features of anxiety disorders in DSM-3, DSM-4, or DSM-5, and all the subjects were assessed for clinical severity through appropriate psychological measurement. (3) Intervention measures: All the studies clearly described the intervention plan and the comparative study between VRCBT and WLG. The study had to specify that an experienced psychotherapist was the one to conduct CBT with patients with anxiety disorders. (4) Result indicator type: In order to be included in this analysis, at least one of the two outcome indicators included in each RCT study had to be anxiety and/or depression symptoms; improvement in the severity of anxiety and depression was measured by an established assessment scale or a scale assessed by a clinical psychologist, and the secondary outcome indicators had to consider the follow-up and withdrawal rate during the intervention. Studies that did not meet the above inclusion criteria were excluded. Disagreements between the two researchers were resolved through discussion with a third reviewer.

### Risk of Bias Within and Across Trials

Two researchers adopted the “bias risk assessment” tool of the Cochrane systematic review to evaluate the quality of six indicators for the included studies: random allocation method, allocation concealment, blinding (investigator-blinded and/or participant-blinded), the integrity of result data, selective reporting of research results, and other sources of bias (21). All studies were scored as possessing (a) low risk of bias, (b) unclear, or (c) high risk of bias (21). Disagreements between researchers were resolved through discussion with a third reviewer.

The GRADE system evaluates the overall quality of the experiment based on the results to provide a transparent and clear interpretation of the study. The table includes five subtraction rules: (a) score of ≤40% on the risk of bias assessment; (b) results between studies are inconsistent; (c) studies used indirect measures to test outcomes; (d) questionable accuracy of data collection; and (e) evidence of publication bias. These rules reduced the overall quality of the study, and so three rules were added: (1) one point for large effect size, two points for very large; (2) evidence of a dose response; and (3) confounding variables were accounted for, which can improve the overall quality of the research. According to the evaluation results, the quality of evidence was divided into four levels: (a) high quality—very confident that the real effect value is close to the effect estimate; (b) medium quality—there is a medium degree of confidence in the effect estimates, and the actual values may be close to the estimated value, but there is still the possibility that the two are different; (c) low quality—the degree of confidence in the estimated value is limited, and the real value may be quite different from the estimated value; (d) low quality—there is little confidence in the effect estimate, and the real value is likely to be very different from the estimated value (22). Evaluations were conducted by one researcher and then checked by another; disagreements between the two researchers were resolved through consensus with a third reviewer.

### Data Extraction

Two researchers conducted data extraction by reading the full text to determine the outcome indicators for analysis independently. The primary outcome indicators included anxiety measurement results and depressive symptoms directly related to the target disease, such as the Social Interaction Anxiety Scale (SIAS) used to evaluate social anxiety treatment effect and the measurement results of Beck Depression Inventory (BDI). The secondary outcome indicators included the rate of midpoint withdrawal and follow-up effects. If a 6-month follow-up (or close follow-up) was reported in the study, we extracted the study's follow-up data. If there were no data needed to calculate the magnitude of the effect of the study, we contacted the author. We also extracted descriptive data, according to the following four aspects: literature characteristics, participant characteristics, intervention plan, and anxiety measurement indicators and test tools. Literature characteristics included author, year of publication, and country. Participant characteristics included types and diagnostic criteria of anxiety disorders (diagnostic tools), number of participants (e.g., VRCBT vs. control group number, and sex ratio), and average age. The intervention plan included weekly dose, duration, and follow-up. Anxiety disorder measurement indicators and test tools included testing tools for anxiety and depression in patients with anxiety disorders.

### Data Analysis

To accurately extract the data, a researcher extracted the data, and a second researcher confirmed the extracted data to ensure accuracy. Using Review Manager 5.3 software for meta-analysis, we adopted the random effect model due to different patients and methodological characteristics regardless of heterogeneity. For continuous data, the standardized mean difference (SMD) was selected as the effect scale index for statistics. The magnitude of effect indicates the degree of influence of VR combined with CBT on anxiety disorders. It is classified as follows: 0.2–0.5 = small effect; 0.5–0.8 = medium effect; >0.8 = large effect (21). The effect values are all expressed in a 95% confidence interval (CI). The heterogeneity between the studies was analyzed using the *I*^2^ statistic, classified as follows: *I*^2^ = 0–24%, low heterogeneity; *I*^2^ = 25–49%, moderate heterogeneity; *I*^2^ = 50–74%, high heterogeneity; *I*^2^ = 75–100%, very large heterogeneity (21). The “leave-one-out” method was used for sensitivity analysis to determine the source of heterogeneity (21). We contacted the authors of studies by email to obtain relevant information for those studies lacking sufficient detail.

## Results

### Studies Reach

**Figure 1:**
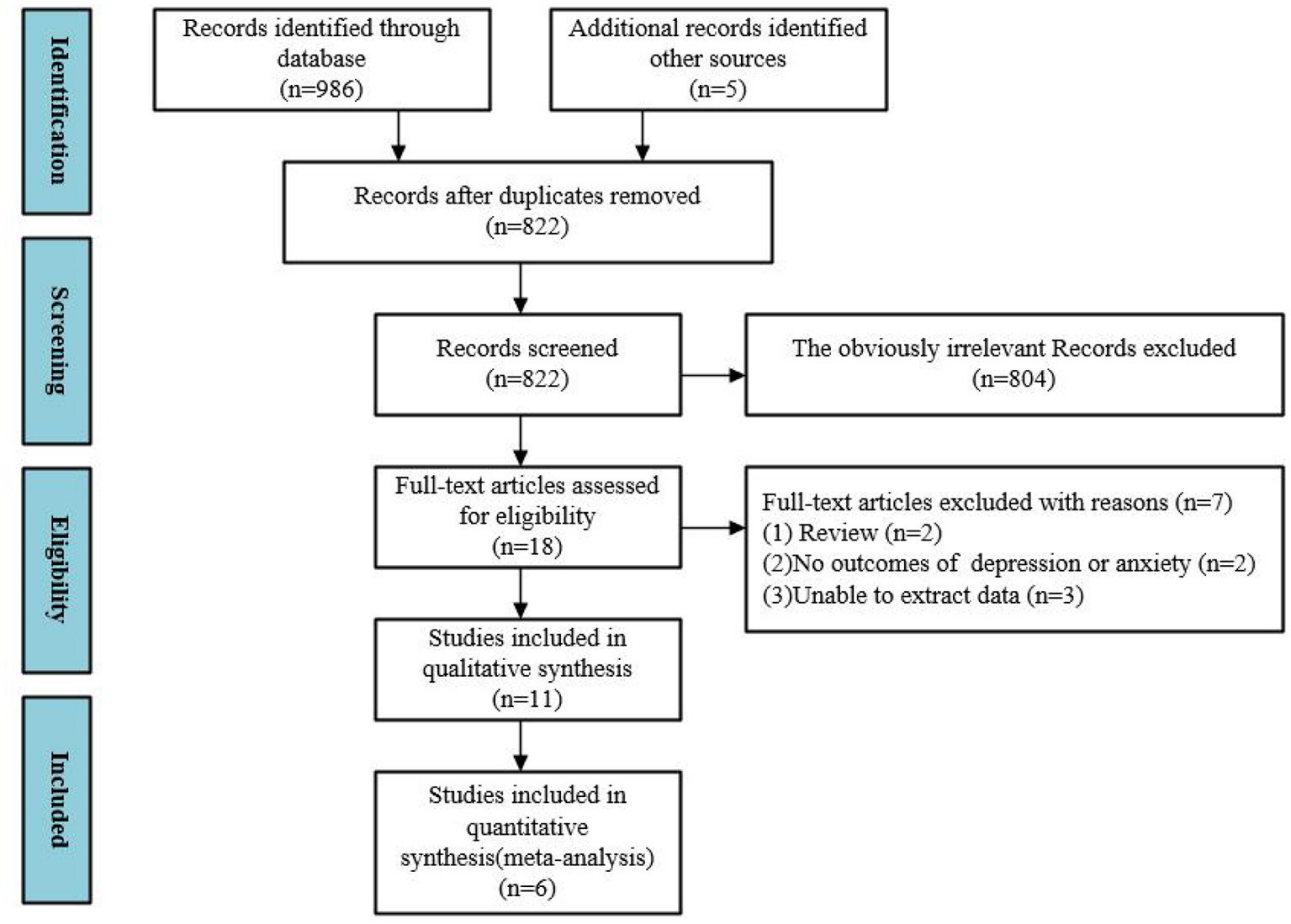
The flow of literature search and study selection.

A summary of the results of the literature search and screening process is shown in Figure 1. A total of 986 records were retrieved, five records were added manually, and the remaining 822 records were removed. A total of 804 records were excluded through the title and abstract, while 18 studies were included in the full-text review. One (23) study concerned VR exposure therapy for phobias, while the other (24) study was a literature overview of VR in treatment of psychiatric disorders. Two (25, 26) studies had no outcome indicators of interest, and three (27, 28) studies did not provide raw data and were therefore excluded. We performed meta-analysis on 6 of these (9, 10, 29–32).

### Risk of Bias

#### Risk of Bias in Individual Studies

**Figure 2:**
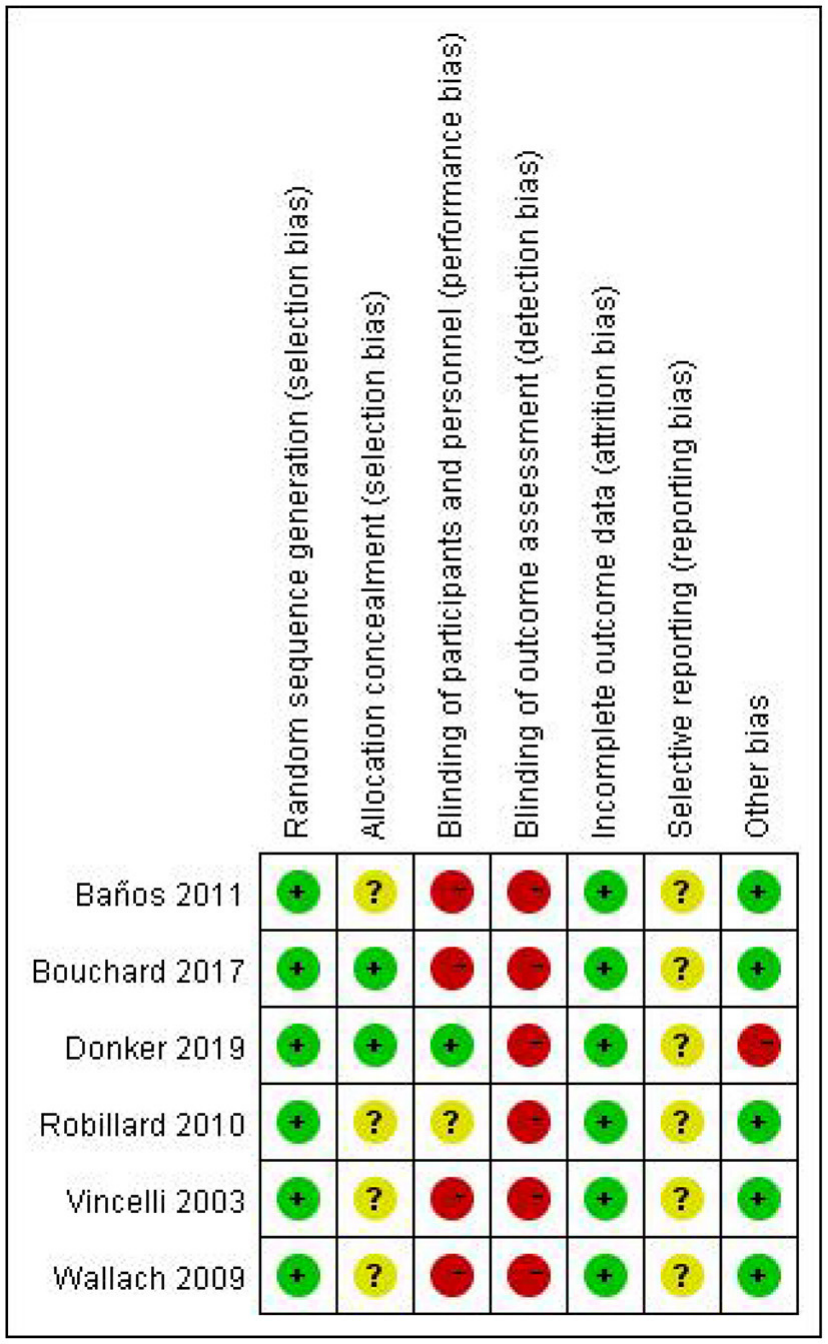
Risk of bias in individual studies. +, low risk of bias; ?, unclear risk of bias; –, high risk of bias.

Results of bias risk assessment are shown in Figure 2. Six (9, 10, 29–32) studies reported the generation of randomized controlled sequences, two studies reported allocation concealment, one (9, 29) study blinded participants, and no studies blinded the evaluation of results. The data of the six (9, 10, 29–32) studies were relatively complete, although, their selective publication was unclear (9, 10, 29–32). One (30) study may have been subject to publication bias due to the use of VR devices with mobile phones as terminals.

#### Grade Assessment

The GRADE assessment results for the overall quality of the experiment are shown in Table 1. We grouped these studies according to different classifications that assessed the quality of evidence. All studies were initially listed as high quality. Based on a high *I*^2^, all groups were removed to indicate inconsistency, and all studies were of medium quality. We have a medium degree of confidence in the effect estimates, and the real value may be close to the estimated value.

**Table 1:** GRADE assessment.

**Outcomes**		**No. of studies**	**No. of participants**	Quality of the Evidence^*^	**Comments**
Anxiety	VRCBT vs. no treatment	5	276	Moderate	Quality reduced due to inconsistent results
Anxiety	VRCBT vs. CBT	4	150	Moderate	Quality reduced due to inconsistent results
Depression	VRCBT vs. no treatment	3	74	Moderate	Quality reduced due to inconsistent results
Depression	VRCBT vs. CBT	4	116	Moderate	Quality reduced due to inconsistent results

* *All studies started with high quality of evidence for being RCTs*.

### Study Characteristics

A summary of all study characteristics is shown in Table 2, where 11 studies on VRCBT treatment of patients with anxiety disorders are summarized and include a total of 6 RCT studies (9, 10, 29–32) published between 2003 and 2019, with a total of 626 patients (age range, 25.3–43.8 years old).

**Table 2:** Study characteristics of eligible studies.

**Reference**		**Participant characteristics**				**Intervention Protocol**		**Measurement**
**Author**	**Country**	**Subjects; diagnostic criteria**	***N***	**M/W**	**Mean age (years)**	**Intervention dose**	**Follow-up (months)**	**Primary outcome**
Vincelli et al. (32)	Italy	Panic disorder; DSM-IV	VRCBT: 4 CBT: 4 WLG: 4	0/12	43.8	VRCBT: 8 sessions of VRCBT therapy CBT: 12 sessions of CBT therapy	Not clear	Anxiety: STAI-S, STAI-T Depression: BDI-2
Anderson et al. (33)	America	Public speaking anxiety; DSM-IV	VRCBT: 8	2/88	Not clear	4 sessions of anxiety management training; 4 sessions of exposure therapy	3	Depression: BDI-2
Wallach et al. (10)	Israel	Public speaking anxiety; structured interview	VRCBT: 45 CBT: 34 WLG: 33	5/23 6/24 9/21	28.2 28.6 25.3	60 h for 12 weeks	12	Anxiety: LSAS-F, LSAS-A
Robillard et al. (31)	Canada	Social anxiety disorder; DSM-IV	VRCBT: 16 CBT: 14 WLG: 15	13/32	34.9	16 sessions of therapy	Not clear	Anxiety: LSAS Depression: BDI-2
Baños et al. (29)	Spain	Posttraumatic stress disorder; pathological grief; adiustment disorders; DSM-IV	VRCBT: 25 CBT: 25	12/27	30.9	Not clear	3	Depression: BDI
Malbos et al. (34)	Australia	Agoraphobia; DSM-IV	VRCBT: 5 VR: 5	Not clear	Not clear	15 h for 8 weeks	Not clear	Anxiety: DASS
Freedman et al. (17)	Israel	Posttraumatic stress disorder; DSM-IV	VRCBT: 50 WLG: 50	Not clear	Not clear	16 sessions of therapy	12	Depression: BDI-2
Moldovan et al. (35)	Not clear	Social phobia; acrophobia; flight phobia; DSM-IV	VRCBT: 16 WLG:16	17/15	Not clear	4 to 5 h for 1 week	Not clear	Anxiety: LSAS
Bouchard et al. (9)	Canada	Social anxiety disorder; DSM-IV	VRCBT: 22 CBT: 17 WLG: 20	7/15 0/17 9/11	36.2 36.7 30.6	60 h for 14 weeks	6	Anxiety: LSAS-SR, SIAS Depression: BDI-2
Donker et al. (30)	Netherlands	Acrophobia; AQ	VRCBT: 96 WLG: 97	64/129	41.3	Not clear	3	Depression: BAI
Geraets et al. (36)	Netherlands	Generalized social anxiety disorder; SIAS >25	VRCBT: 15	7/8	34.9	16-h therapy	6	Anxiety: SIAS Depression: BDI-2

*M/W, men/women; VR+CBT, Virtual reality-assisted cognitive behavioral therapy; CBT, Cognitive behavioral therapy; WLG: Wait-list Group; BDI-2: Beck Depression Inventory 2; STAI-S: State-Trait Anxiety Inventory for Adults-State anxiety scores; STAI-T: State-Trait Anxiety Inventory for Adults-Trait anxiety scores; LSAS-F: Liebowitz Social Anxiety scale-Fear; LSAS-A: Liebowitz Social Anxiety scale-Avoidance; LSAS: Liebowitz Social Anxiety Scale; DASS: Depression Anxiety Stress; LSAS-SR: Liebowitz Social Anxiety Scale-Self Reported version; SIAS: Social Interaction Anxiety Scale; BAI: Beck Anxiety Inventory; SIAS: Social Interaction Anxiety Scale*.

### Data Synthesis

#### Effects of VRCBT on Anxiety

**Figure 3:**
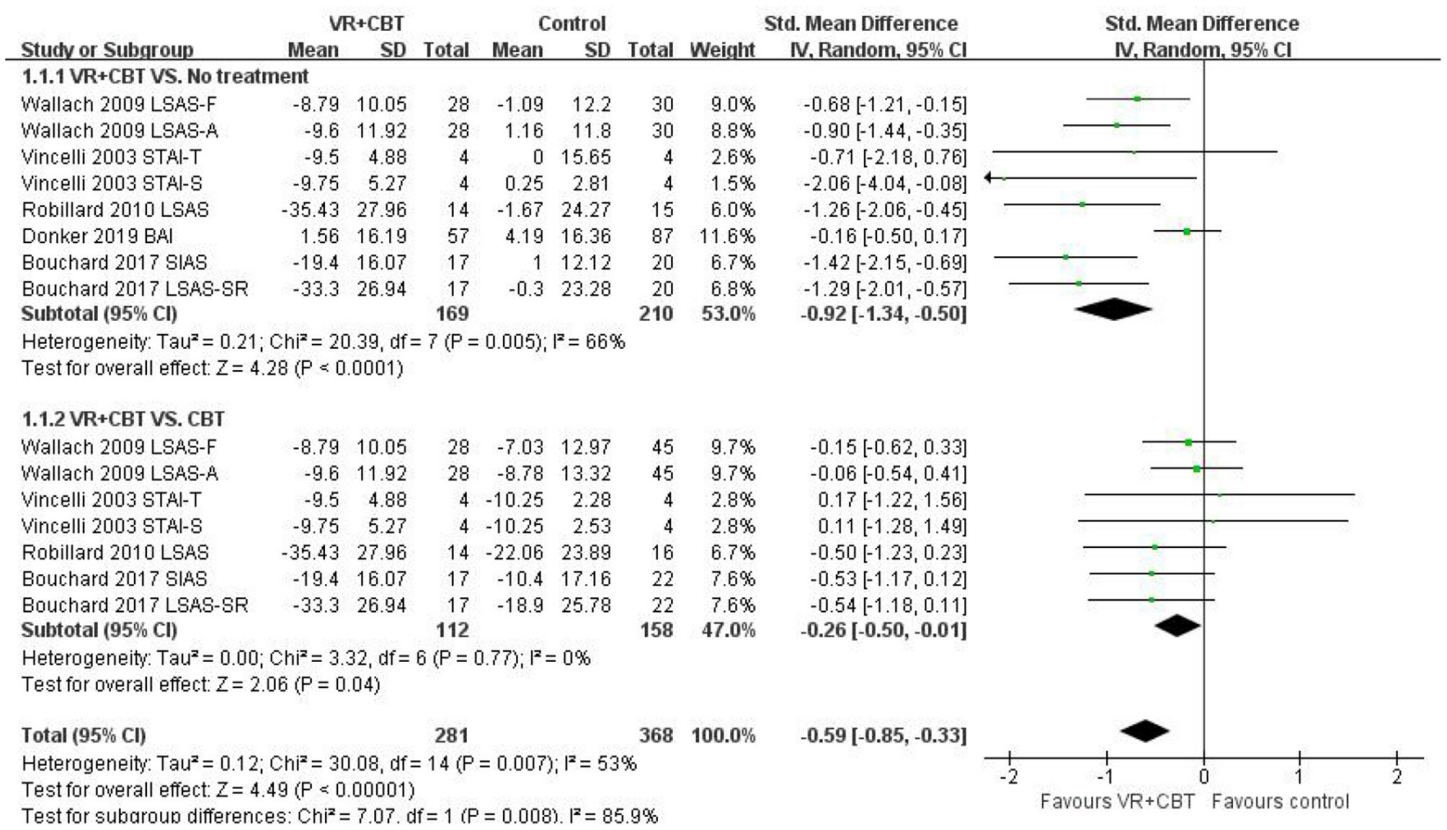
Forest plot of VRCBT vs. no treatment or CBT comparators for anxiety severity.

Meta-analyses revealed evidence of the impact of VRCBT on anxiety. Five (9, 10, 29–32) studies reported a significant effect of VRCBT treatment on the degree of anxiety compared to untreated patients, showing a large effect (SMD = −0.92; 95% CI: −1.34, −0.50; *p* = 0.005, *I*^2^ = 66%; Figure 3), while four studies (9, 10, 31, 32) reported an effect of VRCBT treatment on the degree of anxiety compared to CBT treatment, showing a small effect (SMD = −0.26; 95% CI: −0.50, −0.01; *p* = 0.77, *I*^2^ = 0%; Figure 3). Similarly, there was a group difference in the level of anxiety relief between the untreated group and the CBT-treated group (two RCTs; SMD = −0.59; 95% CI = −0.85, −0.33; *p* = 0.007; *I*^2^ = 53%). A sensitivity analysis of “leave-one-out” was performed on the two groups, and no change in the direction of the effect size was found.

#### Effects of VRCBT on Depression

**Figure 4:**
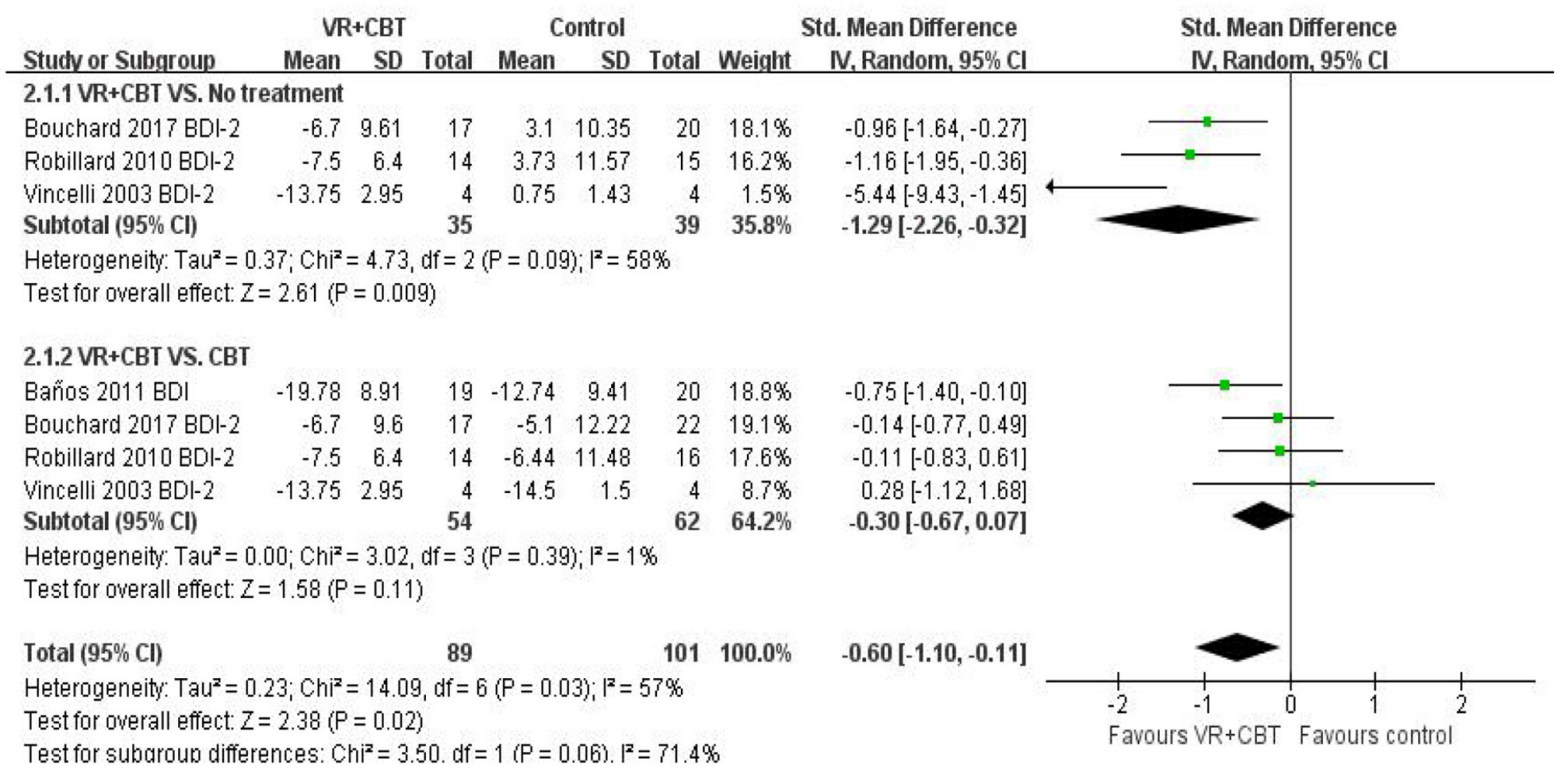
Forest plot of VRCBT vs. no treatment or CBT comparators for depression severity.

Meta-analysis revealed evidence of the effect of VRCBT on depression. Three (9, 31, 32) studies reported the effect of VRCBT treatment on the degree of depression compared to untreated patients (SMD = −1.29; 95% CI: −2.26, −0.32; *p* = 0.09, *I*^2^ = 58%; Figure 4), while four studies (9, 29, 31, 32) reported the effect of VRCBT treatment on the degree of depression compared to CBT treatment (SMD = −0.30; 95% CI:−0.67, −0.07; *p* = 0.39, *I*^2^ = 1%; Figure 4). Similarly, there was a group difference in the level of depression relief between the untreated group and the CBT-treated group (two RCTs; SMD = −0.59; 95% CI = 0.85, −0.33; *p* = 0.007; *I*^2^ = 57%). A sensitivity analysis of “leave-one-out” was performed on the two groups, and no change in the direction of the effect size was found.

#### Dropout Rates

We only compared the dropout rate of VRCBT treatment to that of CBT treatment. Among them, five (9, 10, 29, 31, 32) studies reported the number of dropouts. Due to the relatively small number of patients, only three (9, 10, 29) studies reported withdrawal in the experiment. Examination of dropout rates showed that the dropout rates of the VRCBT group and CBT group did not reach statistical significance (OR = 0.70, 1.48, *p* > 0.05).

#### Long-Term Effects

Of the 11 studies we included, 7 (9, 10, 29, 30) studies followed up patients for 3, 6, and 12 months; however, only 2 (9, 10) studies reported test data for anxiety and depression after follow-up. Since only two studies were included, we did not conduct a meta-analysis on the long-term effectiveness of VRCBT. From the results of these two studies, we found that the effect of the VRCBT group on anxiety and depression levels was maintained after 6 months or 1 year, compared to the post-experiment test, with no statistical significance between VRCBT and standard CBT.

## Discussion

We conducted a meta-analysis on anxiety and depression of anxiety patients treated with VRCBT. This study focused on variables relevant to clinical practice, given the prior established advantages of standard CBT in treating anxiety disorders. We found that the therapeutic effects of VRCBT on anxiety were greater than those of WLG (SMD = −0.92), while the effects of VRCBT were similar to those of standard CBT treatment (SMD = −0.26). We also found that the therapeutic effects of VRCBT on depression were better than those of WLG (SMD = −1.29), while the effects of VRCBT were similar to standard CBT (SMD = −0.30). We further found that although VRCBT and standard CBT did not reach statistical significance in the intervention of anxiety and depression, they showed a positive trend. Due to the insufficient number of existing studies, performing a meta-analysis on follow-up outcomes was incomplete.

We found evidence to suggest that VRCBT has a potential advantage in treating anxiety disorders, as patients can be treated through VR rather than in a real environment (37). In terms of dropout rates, we found no significant significance between VRCBT and standard CBT treatment rates, and of the six studies included, five (9, 10, 29, 31, 32) reported the dropout of VRCBT and CBT in the course of treatment, and only two (9, 10) studies showed a lower VRCBT treatment dropout rate than CBT treatment. These differences are similar to those found in a previous study (38).

In this meta-analysis, we found no overall differences between VRCBT and standard CBT for treating anxiety and depression, but one (9) study did find VRCBT more effective than standard CBT. As VR technology advances and becomes more affordable, we expect an increase in acceptance of this technology.

One (39) study took a survey of patients with social anxiety disorder following the intervention and found that although the levels of anxiety in VRCBT and standard CBT treatment groups were high, the difference between the two groups was not statistically significant. Another study (31) found similar results in patients with social anxiety. During follow-up, the two groups showed similar efficacy. Despite these results, previous studies have suggested that VR exposure therapy may not be enough for anxiety patients and lacks the cognitive components required to treat psychological function; therefore, combining VR with CBT may be an effective step forward (40). Although, the mechanisms of this are unclear, we found evidence that CBT in a virtual environment shows similar efficacy to standard CBT intervention, and these findings have a particular significance for clinical psychologists who may recommend VRCBT for anxiety disorder treatment (41).

VR technology aims to realize human–machine interaction. While experiencing the simulated environment, human sensation and action can be fed back to the computer using sensor technology and stereoscopic display technology. The basic principle of VR technology exposure is that the virtual environment is set up according to the brain emotional network processing model. When a patient confronts a threatening stimulus resulting in a fearful reaction, the fear network is activated. As new and incompatible information is gradually added to the emotional network, habituation and elimination of fear help patients change their fear structure, rendering the stimulus less threatening. During this procedure, the patient must remain under stimulation until anxiety and fear are reduced to a sufficiently low level to achieve the therapeutic effect (42, 43). At present, the new virtual environment can depict a wide range of tasks and more convenient grading exposure, increasing the body's perceived exposure and providing more inhibitory learning for patients, therefore being conducive to recovery (10). For example, when treating patients with social phobia, virtual people can talk and appear in virtual public places, which is an amicable method. Patients with posttraumatic stress disorder can benefit from this therapy by exposure to virtual elements related to traumatic experiences, being encouraged to reflect on their own experiences and feelings (29). Simultaneously, through the therapist's discovery and guidance, we can identify unreasonable cognition in exposure, find ways to replace it, and identify and correct patients' unreasonable cognition, thereby, achieving a therapeutic effect.

Compared to traditional CBT grading exposure, the therapist can expose patients to different levels according to their condition in the VR environment and provide a sense of security for patients. Since VRCBT can be performed in the therapist's office, the VR environment is confidential, and patients do not have to worry about potentially embarrassing situations or privacy concerns. With the continuous updating of VR technology, the procedure is also more straightforward and less expensive compared to traditional treatments. VRCBT is expected to be used in family therapy in the future, saving time and money. Additionally, VRCBT can be used as an intermediate step for patients who refuse to be exposed to reality, which may increase their likelihood of accepting reality exposure through VR exposure.

## Study Limitations

The most important limitation of this study is the limited number of studies included. This study applied a systematic and rigorous search strategy to retrieve relevant articles according to the research objectives. However, studies on VRCBT for anxiety disorders are too rare. It was impossible to conduct subgroup analysis on the efficacy of treatment on different types of anxiety subtypes. It is difficult to give specific recommendations for certain timings and frequency of the interventions. Second, the latest search of this study was conducted on March 3, 2020, and new research findings published after that date were naturally excluded. Third, we only investigated the effects of VRCBT on the cognitive behavior of anxiety patients but did not investigate research related to neural mechanisms of the disorder. Fourth, although, this study has a single intervention, it does not account for the fact that different VR facilities may bring about different effects on anxiety patients. For example, the virtual environment created by computers and mobile phones may be problematic in evaluating efficacy. Fifth, only six studies were included in this study, so the datasets were individually relatively small, resulting in an overall dataset that was similarly small.

## Conclusions

The current meta-analysis shows that VRCBT has a positive effect on reducing anxiety and depression in patients with anxiety disorders. Compared to standard CBT, VRCBT can produce similar therapeutic effects but may provide more timely interventions for anxiety disorders. Future research is needed to confirm the benefits of VRCBT for patients with more diverse types of anxiety disorders.

## Data Availability Statement

The original contributions presented in the study are included in the article/supplementary material, further inquiries can be directed to the corresponding author/s.

## Author Contributions

JW, YS, and ZR contributed to the conception and design of the review. JW, YS, and GZ applied the search strategy. JW, ZZ, and ZR applied the selection criteria. JW and ZR completed assessment of risk of bias. JW and YS analyzed and interpreted the data. JW wrote this manuscript. YS and GZ edited this manuscript. ZR is responsible for the overall project.

## Conflict of Interest

The authors declare that the research was conducted in the absence of any commercial or financial relationships that could be construed as a potential conflict of interest.

## Publisher's Note

All claims expressed in this article are solely those of the authors and do not necessarily represent those of their affiliated organizations, or those of the publisher, the editors and the reviewers. Any product that may be evaluated in this article, or claim that may be made by its manufacturer, is not guaranteed or endorsed by the publisher.
